# Special Issue “Current Management of Early and Advanced Rectal Cancer”

**DOI:** 10.3390/cancers15143574

**Published:** 2023-07-12

**Authors:** Filiberto Belli

**Affiliations:** Colorectal Surgery Unit, Fondazione IRCCS Istituto Nazionale dei Tumori di Milano, 20133 Milan, Italy; filiberto.belli@istitutotumori.mi.it

As expected, surgery for low or ultralow disease remains a challenging issue in rectal cancer treatment. Indeed, achieving sphincter-preserving surgery in this setting is a major goal in terms of quality of life, and three novel approaches to reaching this goal are discussed in the present Special Issue. First, intersphincteric resection (ISR) has expanded the scope of sphincter-preserving surgery to include ultralow rectal cancer. ISR is not a novel technique in rectal cancer surgery, but thanks to the accuracy and gentleness of dissection made possible by robotic platforms, ISR is rapidly establishing itself as an intriguing alternative to abdominoperineal resection in selected cases, as discussed by Piozzi and colleagues in their review [1]. Robotic surgery is one of the most important advances in recent years for rectal cancer surgery, as it allows for a reduction of the risk of involved distal or circumferential margins as well as intraoperative or postoperative complications, as highlighted by Huang et al.’s long-term experience in their multicenter study [2].

The second approach to maximizing the likelihood of sphincter-saving rectal surgery is to increase neoadjuvant treatment by providing both chemotherapy and chemoradiation before surgery to further improve downstaging—this is known as total neoadjuvant therapy (TNT). Indeed, as reported by Falk et al. [3], TNT could provide both increased distant control and a higher pathologic complete response rate, paving the way for organ-sparing procedures, from minimally invasive transanal local excisions to “watch and wait” strategies. However, as Sclafani and colleagues point out, what is currently lacking is the ability to properly select those patients who will benefit from TNT and allow for personalized escalation of neoadjuvant treatments [4]. In other words, considering its possible toxicities, TNT is unlikely to be a “one size fits all” strategy that should be routinely administered to all patients.

The third approach for increasing the rate of sphincter-saving surgery is a change of perspective regarding the role of the distal margin. Clear margins are essential in rectal cancer surgery, but Sorrentino and co-authors [5] demonstrate that a distal margin of 1 mm may be sufficient in selected patients who have a major/complete response after neoadjuvant treatment. Thus, if a distal margin of 1 mm is found on final pathology after surgery for low/ultralow rectal cancer, complete abdominoperineal resection may be avoided in such patients.

Another major point of this Special Issue is the confirmation that rectal cancer treatment is multimodal, with various actors (medical and radiation oncologists, liver surgeons, and reconstructive surgeons) playing roles at different stages of the disease, from early to locally advanced and/or metastatic rectal cancer, as discussed by Wlodarczyk and Lee [6]. As reported by Meldolesi and colleagues [7], treatment of rectal cancer could involve radiation oncologists not only for standard neoadjuvant chemoradiotherapy, but also to deliver a radiotherapy boost on lateral pelvic nodes in cases of clinical involvement at baseline, ensuring improved disease control without surgical excision. In addition, the role of liver surgeons in the multimodal treatment of rectal cancer has grown in recent years, pushing the indication for curative liver resection all the way up to liver transplantation for accurately selected patients with unresectable colorectal liver metastases, as summarized well by Maspero et al. in their review [8]. The multimodal management of rectal cancer includes not only preoperative treatments and surgery, but also adjuvant therapy. Identifying which patients will benefit from adjuvant treatment after neoadjuvant chemoradiation, on the other hand, remains an unmet medical need. Sung et al. shed light on this topic, demonstrating that adjuvant chemotherapy improves overall and recurrence-free survival in patients with perineural invasion and/or a positive surgical margin [9].

A poorly investigated aspect is the enhanced recovery after rectal surgery, which is burdened by the possibility of major complications. Interestingly, Ceresoli et al. reported that low compliance with enhanced recovery protocols is associated with open or long-lasting surgery and advanced age, and it may increase the rate of postoperative complications, implying that there is room for improvement in this setting [10].

Another instance is the management of rectal cancer in patients with familial adenomatous polyposis (FAP). Colletti and colleagues demonstrated that rectal cancer can occur in FAP patients treated by total colectomy and ileo-rectal anastomosis, but it can be easily managed with rectal-saving procedures such as endoscopic resection [11].

Finally, innovations are required for patients for whom sphincter-saving surgery is not possible. Thiel et al. reported their relevant experience with a simplified fasciocutaneous inferior gluteal artery perforator flap for perineal reconstruction after extralevator abdominoperineal resection, demonstrating the feasibility of a tailored approach in reconstructive surgery for a wide perineal defect [12]. 

We are now at the end of this long journey that began two years ago.

This Special Issue was created with the aim of highlighting various issues associated with the multidisciplinary management of this cancer.

We hope to have accomplished this difficult task.

Finally, I would like to thank all of the contributors for their outstanding work and impressive experience in the management and treatment of a still widespread and complex disease.
